# New Photoplethysmographic Signal Analysis Algorithm for Arterial Stiffness Estimation

**DOI:** 10.1155/2013/169035

**Published:** 2013-08-04

**Authors:** Kristjan Pilt, Rain Ferenets, Kalju Meigas, Lars-Göran Lindberg, Kristina Temitski, Margus Viigimaa

**Affiliations:** ^1^Department of Biomedical Engineering, Technomedicum, Tallinn University of Technology, Ehitajate tee 5, 19086 Tallinn, Estonia; ^2^Department of Biomedical Engineering, Linköping Univeristy, 581 85 Linköping, Sweden

## Abstract

The ability to identify premature arterial stiffening is of considerable value in the prevention of cardiovascular diseases. The “ageing index” (*AGI*), which is calculated from the second derivative photoplethysmographic (SDPPG) waveform, has been used as one method for arterial stiffness estimation and the evaluation of cardiovascular ageing. In this study, the new SDPPG analysis algorithm is proposed with optimal filtering and signal normalization in time. The filter parameters were optimized in order to achieve the minimal standard deviation of *AGI*, which gives more effective differentiation between the levels of arterial stiffness. As a result, the optimal low-pass filter edge frequency of 6 Hz and transitionband of 1 Hz were found, which facilitates *AGI* calculation with a standard deviation of 0.06. The study was carried out on 21 healthy subjects and 20 diabetes patients. The linear relationship (*r* = 0.91) between each subject's age and *AGI* was found, and a linear model with regression line was constructed. For diabetes patients, the mean *AGI* value difference from the proposed model *y*_AGI_ was found to be 0.359. The difference was found between healthy and diabetes patients groups with significance level of *P* < 0.0005.

## 1. Introduction

There has been an increased interest in the development of innovative noninvasive methods and devices for the diagnosis of cardiovascular diseases [1–3]. Photoplethysmographic (PPG) waveform analysis has been used as one method [4].

PPG is a noninvasive optical technique for measuring changes in blood circulation that is mainly used for monitoring blood perfusion in the skin. The PPG finger sensor consists of a light emitting diode (LED), which is often red or infrared, and a photodetector (PD) [5]. PD and LED are on the opposite side of the finger. The light is emitted from the LED to the skin and a small fraction of light intensity changes is received by the PD, which are related to blood flow, blood volume, blood vessel wall movement, and the orientation of red blood cells in the underlying tissue [6, 7]. The PPG signal consists of different components: DC and AC components and noise, which can be caused by the poor perfusion state and motion artifacts [5]. Noise can be eliminated by using different filtering techniques [8]. The AC component is synchronous with the heart rate and depends on changes in the pulsatile pressure and pulsatile blood volume.

**Figure 1:**
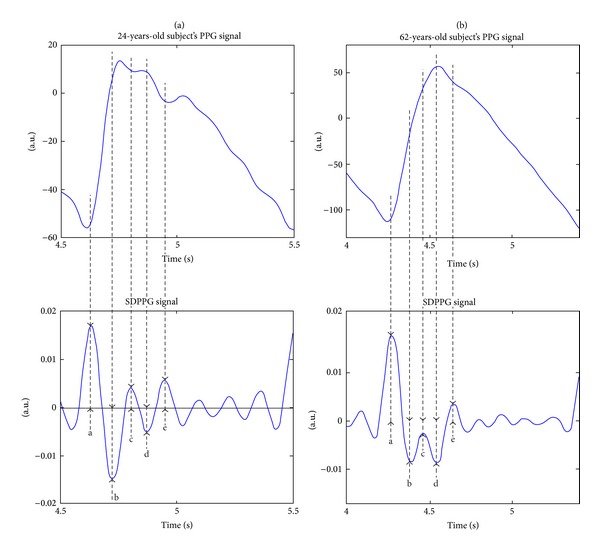
The finger PPG signal and its second derivative with distinct waves “a”, “b”, “c”, “d”, and “e” of 24-year (a) and 62-year-old (b) subjects.

It is apparent that the AC component of the PPG signal changes with age and the waveform transforms from a wavy into a triangular-shaped signal (Figure 1, upper part). Regarding time domain, different methods to analyze the waveform of the PPG signal, measured at the finger, can be used for arterial stiffness estimation and evaluation of cardiovascular aging [9–11].

One option is to use the second derivative of the PPG signal (SDPPG), which was first introduced by Takazawa et al. [12]. The SDPPG is analyzed by using the amplitudes of the distinctive waves “a”, “b”, “c”, “d”, and “e”, which are situated in the systolic phase of the heart cycle (Figure 1, lower part). The amplitudes of the waves are normalized as follows: *b*/*a*, *c*/*a*, *d*/*a*, and *e*/*a*. They found that normalized amplitude *b*/*a* increases and *c*/*a*, *d*/*a*, and *e*/*a* decrease in proportion to the increase in the subject's age. As a result an “ageing index” (*AGI*) parameter was proposed according to *AGI *= (*b*−*c*−*d*−*e*)/*a*, where the *a*, *b*, *c*, *d*, and *e* are the amplitudes of the waves. The *AGI* is used to describe the cardiovascular age of the subject.

In recent publications, the correlation relationship between cardiovascular risk factors and the SDPPG normalized amplitudes values has been analyzed statistically [13–15]. Normalized amplitudes of SDPPG and *AGI* can be good parameters for a screening method to detect increases in the stiffness of the arteries [16].

**Figure 2:**
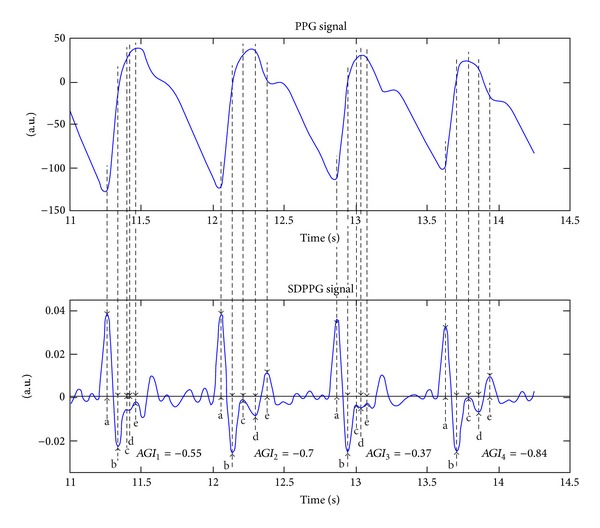
The sample segment of the PPG signal (upper part) from a 37-year-old healthy subject and its second derivative (lower part) with detected wave peaks and *AGI* values. The SDPPG signal is processed, and the wave amplitudes are detected according to a study by Millasseau et al. [17].

The sample segment of PPG and SDPPG signal, which has been registered from a 37-year-old healthy subject, with *AGI* values, is shown in Figure 2. The SDPPG signal is processed, and the wave amplitudes are detected according to a study by Millasseau et al. [17]. The similar processing method has been also described in less detail in other studies [12–15]. It is assumed that the cardiovascular system does not change over short periods in cases of healthy subjects. It is visible from Figure 2 that the *AGI* values for the healthy subject are noticeably higher for the first and third periods. The difference between maximal and minimal *AGI* values is 0.47, which constitutes about 39% from the whole scale of *AGI* [12]. Furthermore, the detected peaks in the first and third periods are located to the beginning of systolic phase of the PPG signal compared to the second and fourth periods. As a result the amplitudes of detected peaks in the consecutive periods describe different phase of the PPG waveform and *AGI* values become noticeably different. This leads to higher standard deviation of *AGI* and to faulty interpretation of the results for a single subject. The detection of the peaks from different phases of PPG signal in case of consecutive periods is due to the insufficient suppression of PPG signal higher components and noise.

The *AGI* has to be calculated with low standard deviation in order to differentiate the subjects with increased stiffness from the healthy subjects. In this study, we have improved the SDPPG analysis method in order to obtain the *AGI* values with minimal standard deviation and to detect the waves at the same locations within one period of the PPG signal. The algorithm is tested on group of healthy subjects and a small group of diabetes patients as a pilot study.

## 2. Methods

### 2.1. SDPPG Analysis Algorithm

**Figure 3:**
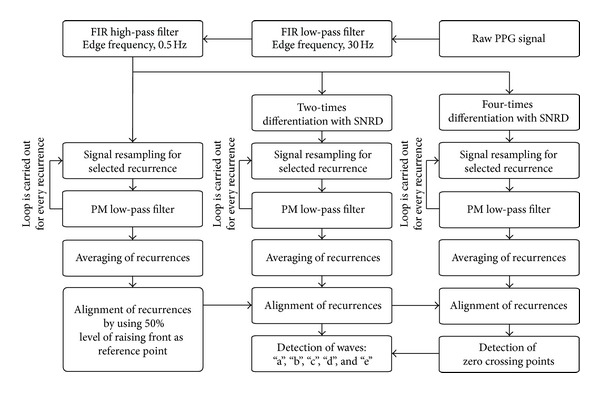
Block diagram of the signal processing for the second derivative analysis.

Normalization of the period's length, averaging, filtering, and detection of the waves from the SDPPG signal are illustrated in a block diagram in Figure 3. The PPG signal is filtered with low- and high-pass FIR filters in order to separate DC components and high frequency noise. The cutoff frequencies for the low- and high-pass filters are selected as 30 Hz and 0.5 Hz, respectively. Both filters are designed using the window method, with the Hamming window function where the corresponding filter orders are chosen as 500 for the low-pass after and 4000 for the high-pass filter.

Subsequently the PPG signal is differentiated two and four times (Figure 3). The simplest differentiator calculates the differences between two consecutive samples of the signal, which is also known as the first-difference differentiator. This kind of differentiator works as a high-pass filter, and the high frequencies are amplified as a result. However, the unwanted noise is located at higher frequencies for the PPG signal. Due to the reason outlined previously, the Smooth Noise Robust Differentiator (SNRD) is used.

The SNRD has been developed for different cases that are particularly beneficial for carrying out experiments with noisy data where differentiation is required [18]. This differentiation scheme possesses the following characteristics: precise at low frequencies, smooth and guaranteed suppression of high frequencies. The order of the differentiator determines the suppression of the high frequencies. In this algorithm, the fifth order of the differentiator is used, which is also the lowest possible one. At the lower frequencies (0–15 Hz), where the majority of the power of the PPG signal is located, the first-difference differentiator and SNRD frequency responses are practically equal.

In practice, biosignals such as PPG, which are related to heart activity, are recurring but not periodic. This means that the harmonic components of the two consecutive recurrences of the PPG signal and its derivatives can be at different frequencies. In this study, the low-pass filter is used with static edge frequency. Accordingly, certain numbers of harmonic components are passed and all others are suppressed. The lengths of the PPG signal recurrences are then normalized to ensure that all the harmonic components are processed in the same way (Figure 3).

The PPG signal is resampled in such a way that one of the selected recurrence lengths is 1 s, which corresponds to the pulse frequency of 1 Hz. In this case, the fundamental frequency is situated at 1 Hz. All the other components lay at the frequency multiples of the fundamental frequency. In the next step, the signal is filtered with the 1 Hz wide transition-band PM filter [19]. The maximum allowable errors for the pass and stop bands are set at 0.001. The resampling and filtering are also carried out with the second and fourth derivatives of the PPG signal. After filtering, the copy of selected recurrence is aligned with other normalized and filtered recurrences from this PPG signal. The recurrences of PPG signals can be aligned by using different distinct points from the signal as reference, for example, the recurrence maximal or minimal point of the raising front. The recurrence minimal point can be difficult to determine, because of the wavy ending of the diastole phase. It is also difficult to determine the PPG signal maximal point as it depends on the state of the cardiovascular system [20]. The 50 percent level of the PPG signal raising front is used as the reference point for the alignment of the recurrences. Furthermore, the second and fourth derivatives are moved according to the movement of the PPG signal recurrences.

**Figure 4:**
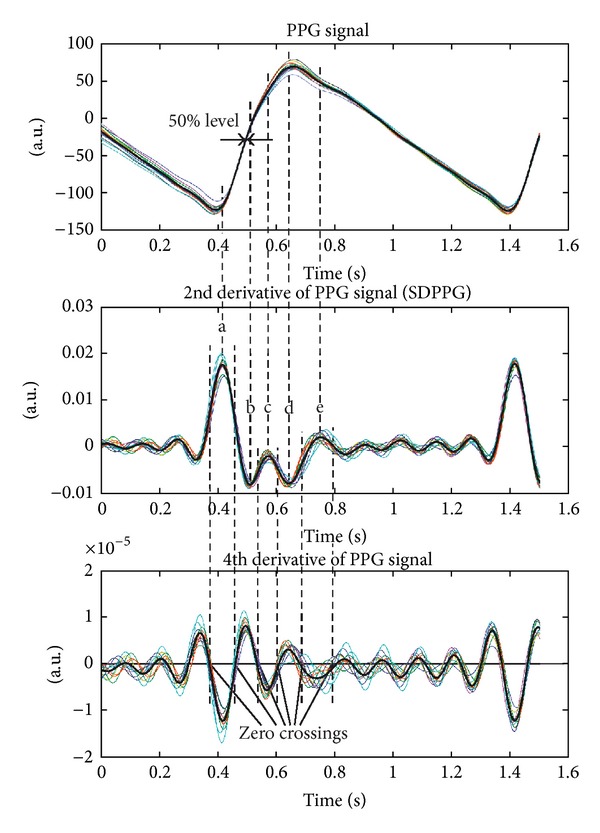
The averaged PPG and its second derivative and fourth derivative waveforms (black bold line) with filtered and normalized recurrences (thin lines). The recurrences are aligned according to 50% of the PPG signal raising front and the distinct waves “a”, “b”, “c”, “d”, and “e” are detected by using the zero crossings of the fourth derivative.

The resampling, filtering, and aligning processes outlined previously are carried out separately for every recurrence in PPG signals. The averaged waveform for one subject with its 9 recurrences is given in Figure 4.

Subsequently, the peaks of waves “a”, “b”, “c”, “d”, and “e” are found from the averaged SDPPG waveform. Firstly, the zero crossings of the averaged fourth derivative waveform are found. The peaks of waves “a”, “b”, “c”, “d”, and “e” are between zero crossings of the fourth derivative waveform as it is revealed in Figure 4. Secondly, the minimal and maximal points of the SDPPG waveform are located between the zero crossings of the fourth derivative waveform. There can be waveforms of the SDPPG, where the peaks of the “c” and “d” waves do not appear. In this case, the “c” and “d” waves are determined in the places of the SDPPG waveform, where the fourth derivative is maximal or minimal between zero crossings.

### 2.2. Optimization of PM Low-Pass Filter Edge Frequency

The recurrences and averaged waveform of the SDPPG are affected by the edge frequency of the PM low-pass filter. The optimal edge frequency of the PM low-pass filter was optimized in order to achieve the lowest standard deviation of the SDPPG wave amplitudes, which ultimately minimizes the standard deviation of *AGI*. In addition, the variation in the placement of waves “a”, “b”, “c”, “d”, and “e” on time domain has to be minimal throughout all the periods for one subject. Here, it is assumed that the cardiovascular system does not change over short periods in cases of healthy subjects. The optimization of the PM low-pass filter edge frequency was carried out on signals from a group of healthy subjects.

The width of the PM low-pass filter transition-band was 1 Hz and the edge frequency was changed between 4 and 14 Hz with a step of 1 Hz. The order of the corresponding PM filter was 3255 at sampling frequency 1 kHz. The 3–13 harmonic components in addition to the fundamental harmonic are passed through the filter as the recurrences of the SDPPG were normalized to the frequency of 1 Hz. In this way, the influence of each harmonic component to waves “a”, “b”, “c”, “d”, and “e” can be analyzed.

The standard deviations for the normalized amplitudes, *b*/*a*, *c*/*a*, *d*/*a*, *e*/*a*, and *AGI* were calculated for each edge frequency. For the standard deviation calculation, the normalized amplitudes, *x*_a_, from normalized SDPPG recurrences and from the averaged SDPPG waveform were used. The normalized amplitudes from the averaged SDPPG waveform were taken as average values *x*_avg_. The standard deviations were calculated for signals from each healthy subject, *k*, by using following equation:

(1) SD(k)=∑i=1n(xa(i)−xavg)2n−1,

where *i* is the number of period and *n* is the total number of periods in the processed signal. Similarly, the standard deviation of the detected wave peaks on the time domain was calculated. The standard deviations were averaged over the group of subjects by using following equation:

(2) SDavg=1m·∑k=1mSD(k),

where *m* is the total number of healthy subjects.

### 2.3. Pilot Study on Patients

The improved SDPPG signal analysis algorithm was tested on the signals from a group of healthy and diabetes patients. The optimal PM low-pass filter edge frequency was used for the analysis. The SDPPG waves were detected and *AGI* values were calculated with standard deviations.

### 2.4. Subjects

The study was performed after approval of the protocol by the Tallinn Ethics Committee on Medical Research at the National Institute for Health Development, Estonia. The PPG signals for the analysis were registered from healthy subjects and diabetes patients.

All the subjects in the healthy group were aged from 21 to 66 years. They were not on permanent medication and they were dealing with various levels of physical activity in their everyday lives. As the waveform of the PPG signal varies with age, the subjects were divided into the following age groups: 20–30, 30–40, 40–50, 50–60, and 60–70. Each age group, except the groups of 50–60 and 60–70, comprised five subjects. Those age groups comprised three subjects, because it was difficult to find healthy subjects to fulfill our criteria. In all, 21 healthy subjects (*m* = 21) participated in the study.

All subjects in the group of diabetes patients had received diagnosis from a medical doctor. In all, 20 diabetes patients participated in this pilot study. The patients were aged between 27 and 66 years. The diabetes patients may have increased arterial stiffness due to the sclerotic processes in the vessels.

### 2.5. Instrumentation

All signal processing was carried out in MATLAB. The high- and low-pass filter coefficients were calculated by using the “fir1” function. A separate function was written for calculating coefficients of the SNRD [18]. The coefficients of PM filter were calculated using functions “firpmord” and “firpm.”

The PPG signals were registered from the index finger by using an experimental measurement complex [21], which included a Nellcor finger clip sensor and lab-built PPG module, among other devices. The PPG signal was digitized with a PCI MIO-16-1 data acquisition card and registered with LabVIEW environment. The sampling frequency was 1 kHz. The 1-minute long signal was recorded, and a 15-period long segment (*n* = 15) was selected for the SDPPG analysis. The signal registration was carried out, while the subject was in a resting position. The subject was in a resting position at least 10 minutes prior to the measurements. The room temperature was around 23 degrees during the experiments.

## 3. Results

**Figure 5:**
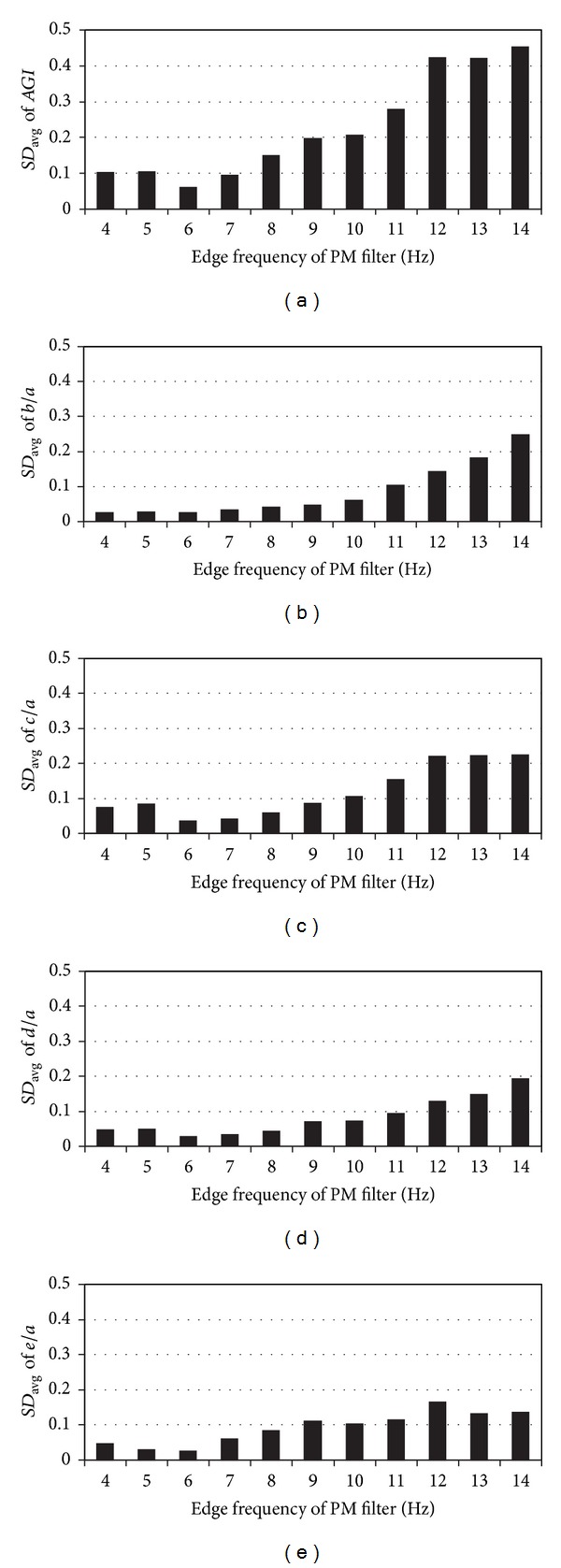
The average standard deviations (*SD*_avg_) of *AGI* and normalized amplitudes at different edge frequencies for 21 healthy subjects. (a) *AGI*s at different edge frequencies; (b) normalized amplitudes *b*/*a* at different edge frequencies; (c) normalized amplitudes *c*/*a* at different edge frequencies; (d) normalized amplitudes *d*/*a* at different edge frequencies; (e) normalized amplitudes *e*/*a* at different edge frequencies.

The general change in standard deviation of the normalized amplitudes and *AGI* in cases of different edge frequencies is illustrated in Figures 5(a)–5(e). For each edge frequency, the given standard deviation is averaged over the group of healthy subjects. The minimal average standard deviation for *AGI*, *b*/*a*, *c*/*a*, *d*/*a*, and *e*/*a* is 0.06, 0.02, 0.04, 0.03, and 0.02 respectively. For all parameters, the minimal standard deviation was found where the edge frequency of the PM filter is 6 Hz and transition band is 1 Hz.

**Figure 6:**
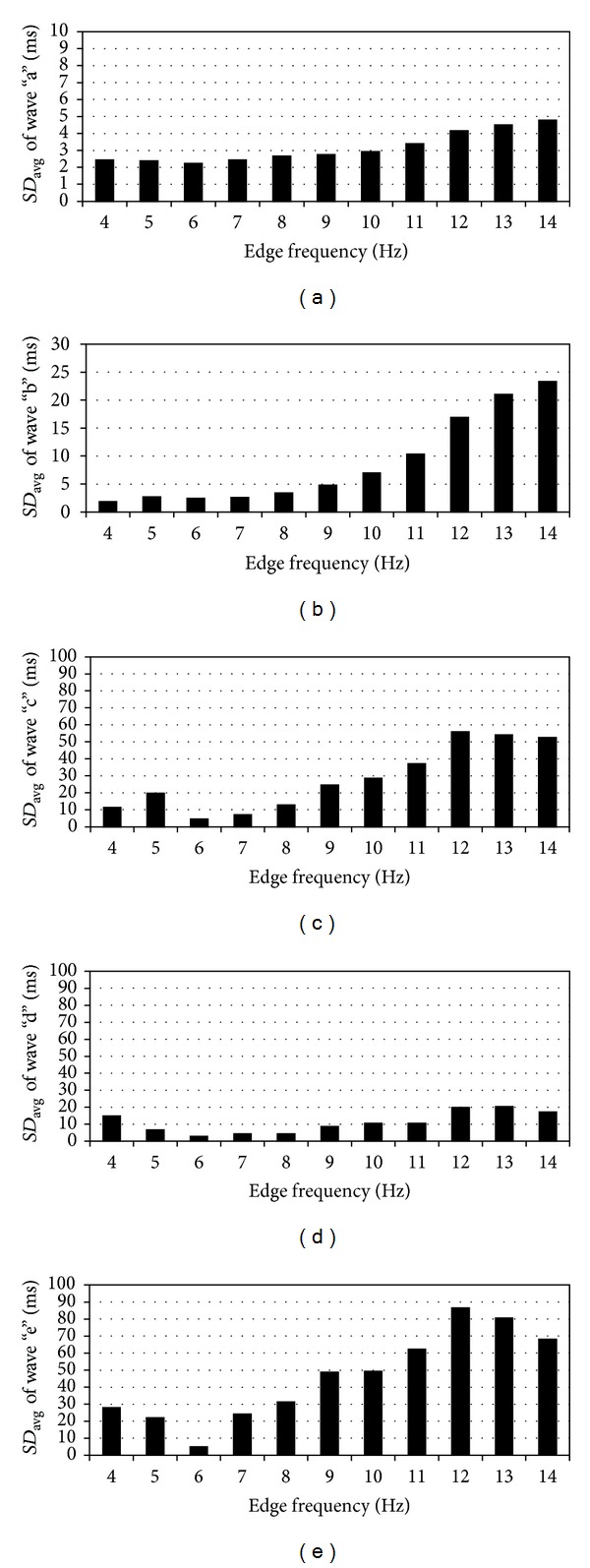
The average standard deviations (*SD*_avg_) of the waves “a”, “b”, “c”, “d”, and “e” on the time domain at different edge frequencies for 21 healthy subjects. (a) standard deviation of wave “a” at different edge frequencies; (b) standard deviation of wave “b” at different edge frequencies; (c) standard deviation of wave “c” at different edge frequencies; (d) standard deviation of wave “d” at different edge frequencies; (e) standard deviation of wave “e” at different edge frequencies.

Similarly, in Figures 6(a)–6(e), the standard deviations to characterize the dispersion of wave peaks “a”, “b”, “c”, “d”, and “e” in the time domain are given. The given standard deviations are averaged over the group of healthy subjects. The minimal average standard deviations for wave peaks “a”, “b”, “c”, “d”, and “e” in the time domain are 2.2 ms, 1.9 ms, 4.6 ms, 2.8 ms, and 5.0 ms, respectively. The minimal standard deviations can be found for the edge frequency of 6 Hz for all waves, except for wave “b”. In the case of wave “b”, the minimal standard deviation was at edge frequency of 4 Hz.

For the purpose of comparison, the same PPG signals were also processed with the algorithm described by Millasseau et al. [17]. The average standard deviation for *AGI* value is 0.12.

**Figure 7:**
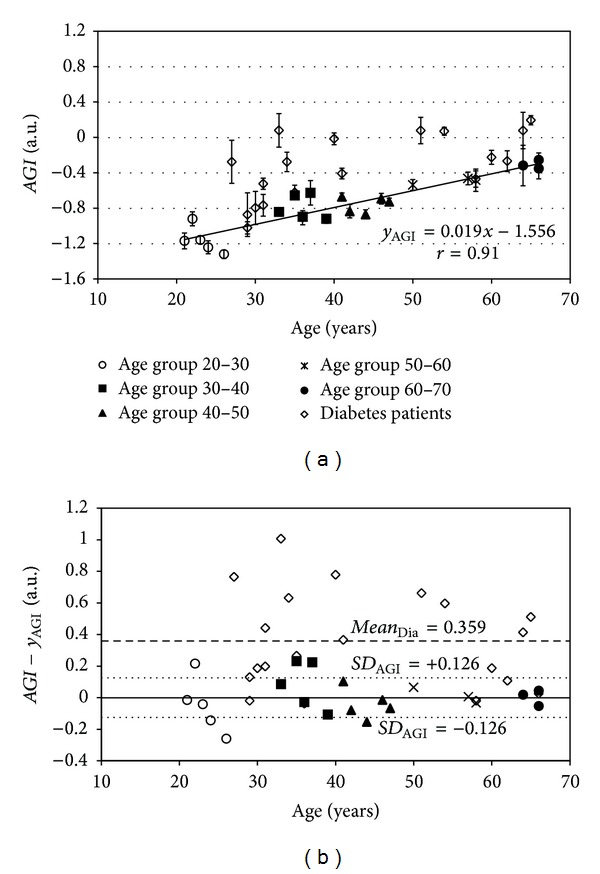
(a) The *AGI* data points with standard deviation (*SD*) bars for groups of healthy subjects and diabetes patients. The linear model line *y*_AGI_ is constructed for a group of healthy subjects. (b) Bland-Altman plot for constructed model. With dotted line the standard deviation levels for group of healthy subjects are given. With dashed line is given the mean *AGI* difference from linear model for diabetes patients.

The *AGI* and age relationship for the healthy subjects and diabetes patients with standard deviation bars are illustrated in Figure 7(a). The PM filter edge frequency and transition-band were 6 Hz and 1 Hz, respectively, which according to the previously presented results seems to be optimal for the SDPPG analysis. In addition, regression analysis was carried out in order to estimate the relationship between *AGI* and age by using generalized linear model. As a first approach the general linear model was used, which is a case of the generalized linear model with identity link function. A following regression model was proposed: *y*_AGI_ = 0.019*x* − 1.556. Despite of relatively simple model high correlation *r* = 0.91 was found between *AGI* and age for the healthy group, which shows the strong linear relationship between two variables.

In Figure 7(b) it can be seen the Bland-Altman plot for the proposed model *y*_AGI_. The standard deviations for the model a *SD*_AGI_ = 0.126. For diabetes patients the mean *AGI* value difference from the proposed model *y*_AGI_ is *mean*_Dia_= 0.359. The *AGI* differences from the proposed model *y*_AGI_ were compared between healthy and diabetes patients groups. Paired *t*-test (two-sample assuming unequal variances) was performed in MS Excel with **α** = 0.05. The significance level of paired *t*-test was *P* < 0.0005, which shows the difference between two groups.

## 4. Discussions

With an improved SDPPG analysis algorithm, the average standard deviation for the *AGI* value is 0.06, which constitutes about 5% of the whole scale of *AGI* [12]. Compared to the algorithm of Millasseau et al. [17], the average standard deviation is twice lower. As a result, subjects with increased arterial stiffness can be more easily differentiated from healthy subjects, and the prevention of cardiovascular disease can be improved.

The relatively high correlation relation was found between *AGI* and age by using the algorithm with optimal edge frequency (Figure 7(a)). This is in relation to previously published results by Takazawa et al. [12], in which a good correlation between *AGI* and age among healthy subjects was shown. There are still some deviations from the regression model line, *y*_AGI_, which can be caused by the impact of cardiovascular deficiencies and the subject's biological age. In addition model can be more complex and dependent on additional variables, such as blood pressure. However, this should be considered in the scope of future studies.

The noticeably higher *AGI* values, compared to the healthy group of subjects, were found for diabetes patients (Figure 7(a)). The same behavior is also visible in Figure 7(b). Furthermore, the statistically significant difference was found between the healthy subjects and diabetes patients. The higher *AGI* values are caused by the increased arterial stiffness of diabetes patients. Nevertheless, some of the diabetes patients have similar *AGI* values compared to healthy subjects. It can be caused by the early diagnosis of diabetes mellitus, which is followed with efficient therapy, and as a result premature stiffening of the arteries has been stopped.

It can be seen from Figures 5(a)–5(e) that the lowest average standard deviation was achieved when the edge frequency of PM filter is 6 Hz. Close to 6 Hz, the normalized recurrences start to resemble. The larger standard deviations on higher edge frequencies are caused by the noise and unwanted frequency components of the PPG signal, which are situated at higher frequencies and amplified through differentiation. This causes the faulty detection of the waves from the single normalized recurrence and averaged SDPPG waveform. At lower edge frequencies, the harmonic components are suppressed, which form waves “a”, “b”, “c”, “d”, and “e”, and the peaks of waves, “c” and “d”, were missing in single normalized recurrences. As a result, the amplitude of the waves in single normalized recurrences and averaged SDPPG waveform are different, which caused the increase in standard deviation (Figures 5(a)–5(e)). This means that it is necessary to have a fundamental harmonic with 5 higher harmonic components in order to detect waves “a”, “b”, “c”, “d”, and “e” from the PPG signal.

The dispersion of wave peaks on time domain decreases, similar to the results seen in Figure 5, when the edge frequency of PM filter approaches 6 Hz (Figures 5(a)–6(e)). As in Figure 5, the detection of the waves from the single normalized recurrences and from the averaged SDPPG waveform can be different at higher frequencies, which increases the standard deviation. At frequencies lower than 6 Hz, the wave peaks can be missing in single normalized recurrence and the detection point is shifted compared with the averaged SDPPG waveform.

## 5. Conclusions

In conclusion, it can be said that the standard deviation of *AGI* values is minimized by using the improved SDPPG algorithm. Furthermore, the diabetes patients have noticeably higher *AGI* values, which are caused by an increase in the arterial stiffness. As a result, the subjects, with increased arterial stiffness can be more easily differentiated from healthy subjects and the prevention of cardiovascular disease can be improved. As a future study the more complex model should be considered in order to enhance the discrimination of the healthy subjects and patients with increased stiffness by taking into account additional physiological variables. In addition proposed algorithm should be compared with similar arterial stiffness estimation reference methods.
